# Triglyceride-Based Indices Versus Homeostasis Model Assessment of Insulin Resistance for Quantitative Insulin Sensitivity Check Index-Defined Insulin Resistance in Metabolic Syndrome: Implications for Resource-Constrained Healthcare

**DOI:** 10.7759/cureus.113851

**Published:** 2026-08-03

**Authors:** Aryan Parida, Rajlaxmi Tiwari, Abhishek P Dash, Chetan K Kellellu, Pragya Panda, Subhashree Ray

**Affiliations:** 1 Biochemistry, Institute of Medical Sciences and Sum Hospital, Bhubaneswar, IND; 2 General Medicine, Institute of Medical Sciences and Sum Hospital, Bhubaneswar, IND

**Keywords:** diagnostic accuracy, homa-ir, insulin resistance, metabolic syndrome, quicki, triglyceride–glucose index (tyg index)

## Abstract

Background

Insulin resistance (IR) is an important pathogenic factor in metabolic syndrome (MetS) and its associated cardiometabolic complications. Insulin-based indices such as the Homeostasis Model Assessment of Insulin Resistance (HOMA-IR) and the Quantitative Insulin Sensitivity Check Index (QUICKI) are well-validated surrogates of IR; however, their dependence on fasting insulin assays restricts their implementation in limited-resource healthcare facilities. Triglyceride-based indices, especially the triglyceride-glucose (TyG) index, have been considered as affordable, insulin-free alternatives. However, their diagnostic accuracy compared with HOMA-IR has been inconsistent across different categories of individuals. Hence, this study aimed to evaluate the incremental efficacy of composite indices comprising anthropometric and glycemic parameters among patients with MetS, and to compare the diagnostic performance of triglyceride-based indices with HOMA-IR for detecting QUICKI-defined IR.

Methodology

This cross-sectional study included 101 participants, comprising 50 healthy controls and 51 participants diagnosed with MetS. Assessments included anthropometric, clinical, and fasting biochemical parameters. The QUICKI index was used to categorize IR. Triglycerides, the TyG index, fasting insulin, HOMA-IR, and composite indicators (body mass index (BMI) + glycated hemoglobin (HbA1c)), TyG + BMI, TyG + BMI + HbA1c) were all subjected to receiver operating characteristic curve analysis. Diagnostic odds ratios (DORs), sensitivity, specificity, and area under the curve (AUC) were calculated at cut-offs derived using the Youden Index. To determine the independent variables associated with IR as determined by QUICKI, multivariate logistic regression was used.

Results

Participants with MetS exhibited substantially lower QUICKI scores (p < 0.001) and significantly higher BMI, waist circumference, blood pressure, fasting insulin, HOMA-IR, HbA1c, and TyG-based parameters. The differentiation efficacy of HOMA-IR (AUC = 0.978) and fasting insulin (AUC = 0.972) was relatively high. The diagnostic effectiveness of triglycerides alone was inadequate (AUC = 0.572). Strong diagnostic accuracy was achieved using composite indices, specifically BMI + HbA1c (AUC = 0.868) and TyG + BMI + HbA1c (AUC = 0.874), with high specificity (91.7%) and high DORs (132) among the MetS subgroup. Triglycerides were not independently significant (p = 0.121), whereas BMI (odds ratio (OR) = 1.257; p = 0.002) and HbA1c (OR = 20.552; p < 0.001) were independent predictors of IR on multivariate analysis.

Conclusions

Compared with HOMA-IR, independent triglyceride-based indices show inadequate diagnostic significance for identifying QUICKI-defined IR. However, composite indices that include BMI, HbA1c, and lipid characteristics provide reliable, effective substitutes that are not dependent on insulin assays, which makes them suitable for medical facilities with limited resources. These results strengthen a multi-parameter approach to scalable IR screening and risk assessment in MetS.

## Introduction

Central obesity, dyslipidemia, dysglycemia, and hypertension are among the interlinked cardiometabolic disorders causing metabolic syndrome (MetS), which significantly increases the risk of type 2 diabetes mellitus (T2DM) and cardiovascular disease [1]. Insulin resistance (IR), a pathophysiological complication characterized by decreased cellular response to insulin that causes compensatory hyperinsulinemia, increasing glycemic dysregulation, and multiorgan metabolic dysfunction, represents the core of this syndrome [2]. Timely and proper diagnosis of IR is important for both clinical and public health, as it contributes to the development of cardiometabolic complications and increases the risk of T2DM and cardiovascular disease, and is beneficial for early lifestyle and pharmaceutical therapy [2,3].

For the evaluation of IR in clinical and population-based studies, several substituted indices have been developed. Because of its ease of use and robust association with gold-standard methods such as the hyperinsulinemic-euglycemic clamp, the Homeostatic Model Assessment for Insulin Resistance (HOMA-IR), which is derived from fasting insulin and fasting glucose, was the most preferred among these [4]. Similar to fasting insulin and glucose, the Quantitative Insulin Sensitivity Check Index (QUICKI) provides a continuous indicator of insulin sensitivity and has been established as a reliable reference tool for classifying IR status [5]. In a variety of demographics and research settings, both indices have been shown to perform effectively [6]. Being dependent on fasting serum insulin measurement, a cost-effective method considered challenging to perform, subject to multidisciplinary variability, and often inaccessible in peripheral and resource-limited healthcare facilities, especially in low- and middle-income countries (LMICs), is a major drawback common to both methods [7,8].

Triglyceride-based indices, specifically the triglyceride-glucose (TyG) index, have emerged as beneficial, insulin-free alternatives for evaluating IR in response to these limitations [9,10]. The TyG index is calculated by making use of the established pathophysiological association between hypertriglyceridemia, impaired lipid metabolism, and hepatic IR. It is determined as the natural logarithm of the product of fasting triglycerides and fasting glucose divided by two [11,12]. The TyG index is promising for large-scale screening in settings with limited resources because it is intrinsically accessible and economical due to its complete dependence on parameters commonly assessed in standard metabolic panels [11]. The TyG index has been shown in several studies to have moderate-to-good diagnostic performance for MetS and IR detection, with area under the curve (AUC) values varying widely between groups [9,13]. However, its diagnostic accuracy in relation to HOMA-IR remains inconsistent, and comparative performance may be correlated with population-specific metabolic profiles, obesity indices, and insulin metabolism [8,10].

To overcome the limitations of single-parameter markers, composite indices that incorporate many aspects of metabolic dysfunction, including anthropometric, glycemic, and lipid parameters, have been proposed recently. Theoretically, indices that combine the TyG index with anthropometric measurements or body mass index (BMI) with glycated hemoglobin (HbA1c) can represent a wider pathophysiological spectrum of IR than any single marker alone [14,15]. BMI, a stand-in for adiposity-driven metabolic dysfunction, and HbA1c, which represents chronic glycemic exposure over about three months, complement biological aspects of IR that are not considered in lipid-only indices [14]. However, there is relatively limited reliable evidence that statistically validates these composite indices versus QUICKI-defined IR in populations with MetS, especially in South Asian and Indian cohorts [16,17].

According to a distinctive combination of genetic susceptibility, the “thin-fat” adiposity phenotype, fast urbanization, and dietary shift, MetS and its consequences are disproportionately prevalent in India [18,19]. However, the healthcare system in the country frequently lacks the laboratory capacity for insulin assays, particularly at the primary and secondary care levels, which creates a structural gap in the capability to screen and evaluate cardiometabolic risk at scale [20,21]. In context with this, there is a critical need for verified, reasonably priced, and scalable IR evaluation methods [22,23]. While regional studies have started looking at alternative IR markers in South Asian populations, there are still few comparative analyses that specifically evaluate how well triglyceride-based and composite indices perform against QUICKI-defined IR in a characterized Indian MetS population [16,17,23].

To identify QUICKI-defined IR in individuals with and without MetS, the current study aims to systematically assess and compare the diagnostic performance of triglyceride-based indices, such as serum triglycerides and the TyG index, with HOMA-IR and fasting insulin. The study additionally investigates whether composite indices including BMI, HbA1c, and TyG-derived components could improve diagnostic accuracy in a manner considered clinically beneficial, with an emphasis on healthcare facilities with limited resources.

## Materials and methods

Study design and setting

This cross-sectional observational study was conducted in the Department of Biochemistry in collaboration with the Department of General Medicine at IMS and SUM Hospital, Bhubaneswar, Odisha, India. The Institutional Ethics Committee (approval number: Ref. no/IEC/IMS.SH/SOA/2023/623) approved the study. The study was conducted as a part of the short-term studentship project in accordance with the norms of the Indian Council of Medical Research. Written informed consent was obtained from all participants before enrolment.

Study participants

The study included 101 participants, which included 50 healthy participants (controls) and 51 participants with diagnosed MetS (cases). During the study period, participants were recruited from the outpatient departments of endocrinology and medicine. Participants were enrolled according to the eligibility criteria summarized in Table 1.

**Table 1:** Eligibility criteria. JIS: Joint Interim Statement; eGFR: estimated glomerular filtration rate

Inclusion criteria	Exclusion criteria
Adults aged 18–65 years	Pregnancy or lactation
Both sexes	Type 1 diabetes mellitus
Written informed consent	Chronic liver disease
Metabolic syndrome diagnosed according to the JIS criteria	Chronic kidney disease (eGFR <60 mL/minute/1.73m²)
Apparently healthy controls	Thyroid dysfunction
	Active infection
	Drugs affecting glucose/lipid metabolism
	Refusal to participate

Sample size

The sample size was determined based on the anticipated AUC of the TyG index for IR detection, informed by prior regional literature [9,23]. Using a minimum detectable AUC of 0.75 with a significance level of 0.05 and a power of 80%, and accounting for a 10% dropout or exclusion rate, a minimum of 45 participants per group was estimated to be required. A final sample of 51 cases and 50 controls was enrolled to ensure adequate statistical power.

Anthropometric and clinical assessment

All anthropometric and clinical measurements were performed by trained personnel using standardized techniques following a uniform protocol. Body weight was measured to the nearest 0.1 kg using a calibrated digital weighing scale with participants wearing light clothing and no footwear. Standing height was measured to the nearest 0.1 cm using a wall-mounted stadiometer. BMI was calculated as weight in kilograms divided by the square of height in meters (kg/m²). Waist circumference (WC) was measured to the nearest 0.1 cm at the midpoint between the inferior costal margin and the iliac crest at the end of normal expiration using a non-stretchable measuring tape. Blood pressure was measured in the right arm after the participant had been seated quietly for at least five minutes using a calibrated mercury or aneroid sphygmomanometer with an appropriately sized cuff. Two blood pressure measurements were obtained at five-minute intervals, and the mean of the two readings was used for statistical analysis.

Biochemical investigations

Venous blood samples were collected after a minimum of 10-12 hours of overnight fasting. Samples were processed and analyzed in the institutional biochemistry laboratory using standardized, quality-controlled analytical methods. The following biochemical parameters were measured: fasting plasma glucose (FPG), serum insulin, total cholesterol, triglycerides, high-density lipoprotein-cholesterol (HDL-C), and low-density lipoprotein-cholesterol (LDL-C), which were analyzed using the Cobas Pro Integrated Solutions (Roche Diagnostics) chemistry and immunoassay autoanalyzer. HbA1c levels were determined using the D-10 Hemoglobin Testing System (Bio-Rad Laboratories) via high-performance liquid chromatography. The internal and external quality control for the mentioned parameters was maintained in accordance with Clinical and Laboratory Standards Institute guidelines.

Calculation of Metabolic Indices

The following indices were calculated from measured fasting parameters:

HOMA-IR:

\(\mathrm{HOMA\text{-}IR} = \frac{\text{Fasting insulin }(\mu\mathrm{IU}/\mathrm{mL}) \times \text{Fasting glucose }(\mathrm{mmol}/\mathrm{L})}{22.5}\)

QUICKI:

\(\mathrm{QUICKI} = \frac{1} {\log_{10}\!\left(\text{Fasting insulin }(\mu\mathrm{IU}/\mathrm{mL})\right) + \log_{10}\!\left(\text{Fasting glucose }(\mathrm{mg}/\mathrm{dL})\right)}\)

Participants were classified by QUICKI score into the following three categories: insulin sensitive (QUICKI > 0.382), borderline IR (QUICKI 0.332-0.382), and insulin resistant (QUICKI < 0.332), based on established thresholds [5,7]. For binary diagnostic analysis, participants were categorized as insulin sensitive (QUICKI > 0.382) versus borderline or insulin resistant (QUICKI ≤ 0.382).

TyG Index:

\(\mathrm{TyG} = \ln \left[ \frac{\text{Fasting triglycerides }(\mathrm{mg}/\mathrm{dL}) \times \text{Fasting glucose }(\mathrm{mg}/\mathrm{dL})}{2} \right]\)

Composite Indices

Three composite indices were constructed using logistic regression-derived predicted probability scores incorporating the parameter combinations listed in Table 2.

**Table 2:** Composite indices. BMI: body mass index; HbA1c: glycated hemoglobin; TyG: triglyceride-glucose

Composite index	Components
TyG + BMI	TyG index and BMI
BMI + HbA1c	BMI and HbA1c
TyG + BMI + HbA1c	TyG index, BMI, and HbA1c

Each composite score was derived as the predicted probability from a binary logistic regression model with QUICKI-defined IR status as the outcome variable, fitted on the study sample. The resulting probability score (0-1) was used as the continuous predictor in subsequent receiver operating characteristic (ROC) curve analysis.

Definition of metabolic syndrome

MetS was diagnosed according to the 2009 Joint Interim Statement (JIS) consensus criteria [1], requiring the presence of any three or more of the five diagnostic components summarized in Table 3.

**Table 3:** Metabolic syndrome was diagnosed according to the Joint Interim Statement criteria. HDL: high-density lipoprotein

Criterion	Definition
Waist circumference	≥90 cm (men), ≥80 cm (women)
Fasting glucose	≥100 mg/dL
Triglycerides	≥150 mg/dL
HDL cholesterol	<40 mg/dL (men)/<50 mg/dL (women)
Blood pressure	≥130/85 mmHg

Statistical analysis

Data were entered into Microsoft Excel (Microsoft Corp., Redmond, WA, USA) and analyzed using SPSS Statistics for Windows, version 26.0 (IBM Corp., Armonk, NY, USA). Continuous variables were assessed for normality using the Kolmogorov-Smirnov test and are presented as mean ± standard deviation (SD). Categorical variables were compared using Pearson’s chi-square test; test statistics are reported with degrees of freedom and sample size, with phi (2 × 2) or Cramer’s V (larger tables) as effect size, and odds ratios (ORs) with 95% confidence intervals (CIs) for 2 × 2 comparisons. No cell had an expected count below five, so continuity correction and exact tests were not required (Yates-corrected values given for completeness). A two-tailed p-value <0.05 was considered statistically significant.

Diagnostic performance was evaluated using ROC curve analysis, with QUICKI-defined IR (insulin-sensitive vs. borderline/insulin-resistant) as the reference outcome. The area under the ROC curve (AUC) with 95% CIs, sensitivity, specificity, positive predictive value (PPV), negative predictive value (NPV), and diagnostic odds ratio (DORs) were calculated at the optimal cut-off value determined using the Youden Index (J = sensitivity + specificity − 1). ROC analyses were performed for the overall study population as well as separately for the control and MetS groups to evaluate subgroup-specific diagnostic performance. Differences between correlated ROC curves were compared using the DeLong method.

Binary logistic regression analysis was performed to identify independent predictors of QUICKI-defined IR. Serum triglycerides, BMI, and HbA1c were entered into the model as independent variables, while QUICKI-defined IR status served as the dependent variable. The regression results are presented as regression coefficients (B), ORs, 95% CIs, and corresponding p-values.

## Results

A total of 101 participants were enrolled in the study, of whom 51 were diagnosed with MetS and 50 were controls. Table 4 presents the baseline demographic, anthropometric, and biochemical characteristics of the study population. In comparison to controls, subjects with MetS were substantially older and had higher adiposity metrics, such as BMI and WC, in addition to increased blood pressure levels. From a metabolic perspective, patients exhibited markedly elevated fasting insulin, HOMA-IR, HbA1c, and TyG-based indices, with a considerably reduced QUICKI score, signifying compromised insulin sensitivity. Notably, while triglyceride levels were quantitatively elevated in cases, they did not differ significantly across groups, indicating low discriminatory utility at the group-comparison level. Table 4 depicts a distinct metabolic gradient across groups, with indicators of IR showing the most significant differences.

**Table 4:** Distribution and comparison of study parameters using the chi-square and Student’s t-tests. A p-value <0.05 is considered statistically significant. BMI: body mass index; HbA1c: glycated hemoglobin; HDL: high-density lipoprotein; HOMA-IR: Homeostatic Model Assessment for Insulin Resistance; LDL: low-density lipoprotein; QUICKI: Quantitative Insulin Sensitivity Check Index; TyG: triglyceride-glucose

		Normal (N = 50)	Metabolic syndrome (N = 51)	P-value
Sex	Male	28 (56%)	23 (45.1%)	0.273
	Female	22 (44%)	28 (54.9%)	
Age (years)		36.04 ± 10.92	45.18 ± 12.81	<0.001
Height (cm)		166.86 ± 8.63	158.39 ± 12.59	<0.001
Weight (kg)		65.91 ± 7.99	79.76 ± 18.22	<0.001
BMI (kg/m^2^)		23.66 ± 2.06	32.11 ± 8.67	<0.001
Waist (in cm)		93.23 ± 8.26	107.51 ± 8.87	<0.001
Systolic blood pressure (mmHg)		112.84 ± 8.17	139.57 ± 16.71	<0.001
Diastolic blood pressure (mmHg)		76.3 ± 5.07	88.71 ± 8.19	<0.001
Total cholesterol (mg/dL)		104.24 ± 20.	210.31 ± 50.1	<0.001
Triglycerides (mg/dL)		177.12 ± 28.67	194.2 ± 105.62	0.272
HDL (mg/dL)		47.67 ± 7.72	44.94 ± 14.13	0.232
LDL (mg/dL)		47.67 ± 7.72	124.2 ± 38.4	<0.001
HbA1c (%)		5.31 ± 0.35	6.05 ± 0.74	<0.001
Fasting blood sugar (mg/dL)		105.41 ± 28.28	107.16 ± 23.13	0.734
Fasting insulin (µIU/mL)		7.88 ± 4.29	17.79 ± 8.58	<0.001
HOMA-IR		1.84 ± 1.09	4.9 ± 3	<0.001
TyG index		8.46 ± 0.28	9.13 ± 0.48	<0.001
QUICKI score		0.36 ± 0.05	0.31 ± 0.03	<0.001
QUICKI class	Insulin sensitive	36 (72%)	12 (23.53%)	<0.001
	Borderline	13 (26%)	20 (39.22%)	
	Insulin resistant	1 (2%)	19 (37.25%)	
Insulin sensitivity	Insulin sensitive	36 (72%)	12 (23.53%)	<0.001
	Borderline and resistant	14 (28%)	39 (76.47%)	

The results of the chi-square analyses for categorical variables are summarized in Table 5. Sex distribution did not differ between groups (χ²(1, N = 101) = 1.20, p = 0.273; φ = 0.11; OR = 1.55, 95% CI = 0.71-3.40). QUICKI-defined insulin sensitivity class differed significantly across groups (χ²(2, N = 101) = 29.68, p < 0.001; Cramer’s V = 0.54, large effect). When dichotomized, MetS patients were markedly more likely to be borderline or insulin resistant (χ²(1, N = 101) = 23.79, p < 0.001; φ = 0.49; OR = 8.36, 95% CI = 3.42-20.44).

**Table 5:** Chi-square analysis of categorical variables between the control and metabolic syndrome groups. Odds ratios with 95% confidence intervals are reported for 2 × 2 comparisons. A p-value <0.05 was considered statistically significant. χ²: Pearson chi-square statistic; df: degrees of freedom; N: total sample size; φ: phi coefficient (effect size for 2 × 2 contingency tables); Cramer’s V: effect size for contingency tables larger than 2 × 2; OR: odds ratio; CI: confidence interval; QUICKI: Quantitative Insulin Sensitivity Check Index.

Variable	χ² (df, N)	Effect size	OR (95% CI)	P-value
Sex	1.20 (1, 101)	φ = 0.11	1.55 (0.71–3.40)	0.273
QUICKI classification	29.68 (2, 101)	Cramer’s V = 0.54	—	<0.001
Insulin sensitivity	23.79 (1, 101)	φ = 0.49	8.36 (3.42–20.44)	<0.001

In a multivariate binomial analysis of triglycerides, the parameters with the best model for targeting IR, as indicated by QUICKI, BMI, and HbA1c, were identified. The results of the analysis are presented in Table 6.

**Table 6:** Multivariate analysis for QUICKI with triglycerides. BMI: body mass index; CI: confidence interval; HbA1c: glycated hemoglobin; OR: odds ratio

Parameter	B (regression coefficient)	OR	95% CI	P-value
Triglycerides	0.011	1.011	0.997–1.026	0.121
BMI	0.228	1.257	1.089–1.450	0.002
HbA1c	3.023	20.552	3.861–109.041	<0.001

**Figure 1:**
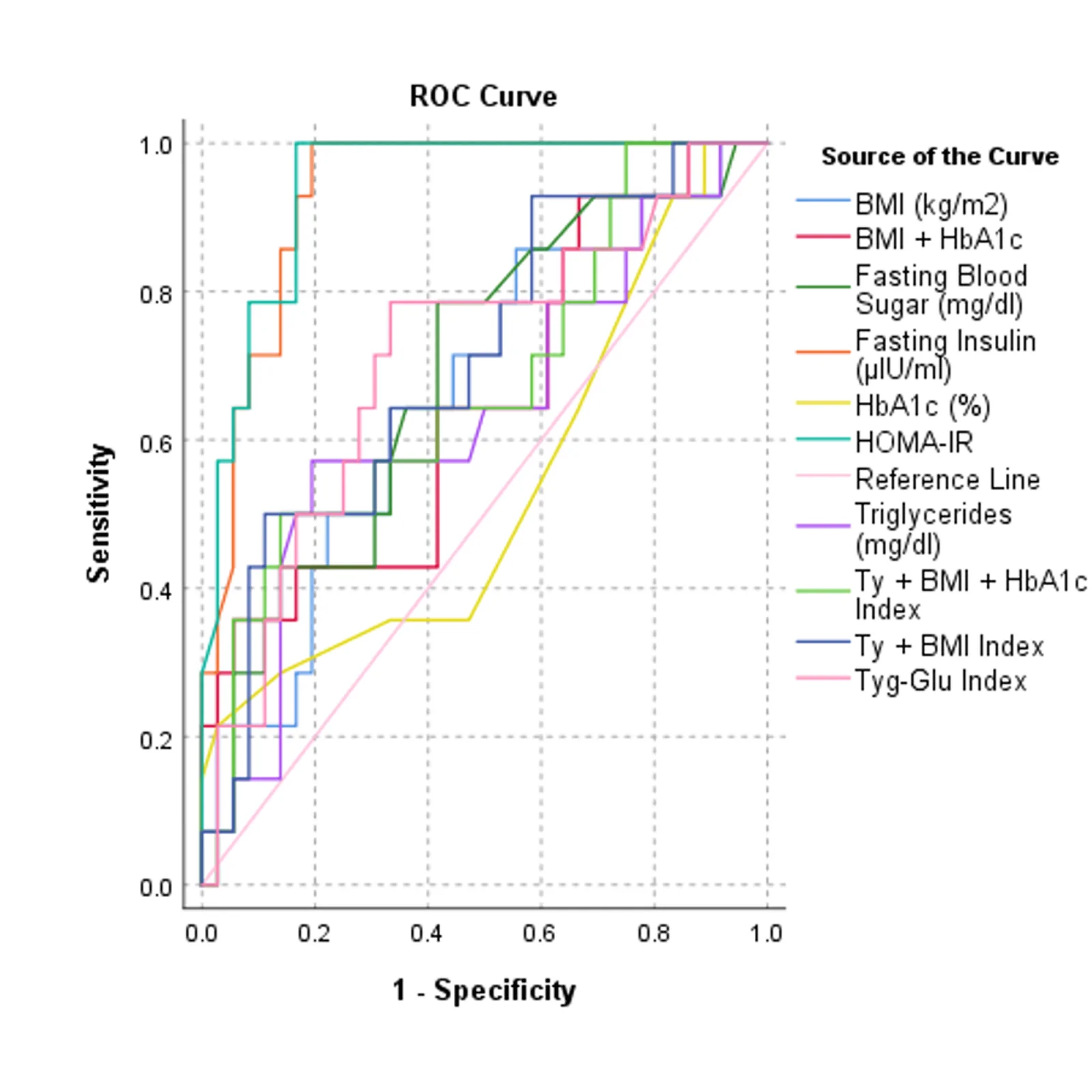
Diagnostic power of various parameters in the control group. ROC: receiver operating characteristic; BMI: body mass index; HbA1c: glycated hemoglobin; HOMA-IR: Homeostasis Model Assessment of Insulin Resistance; TyG: triglyceride-glucose

**Figure 2:**
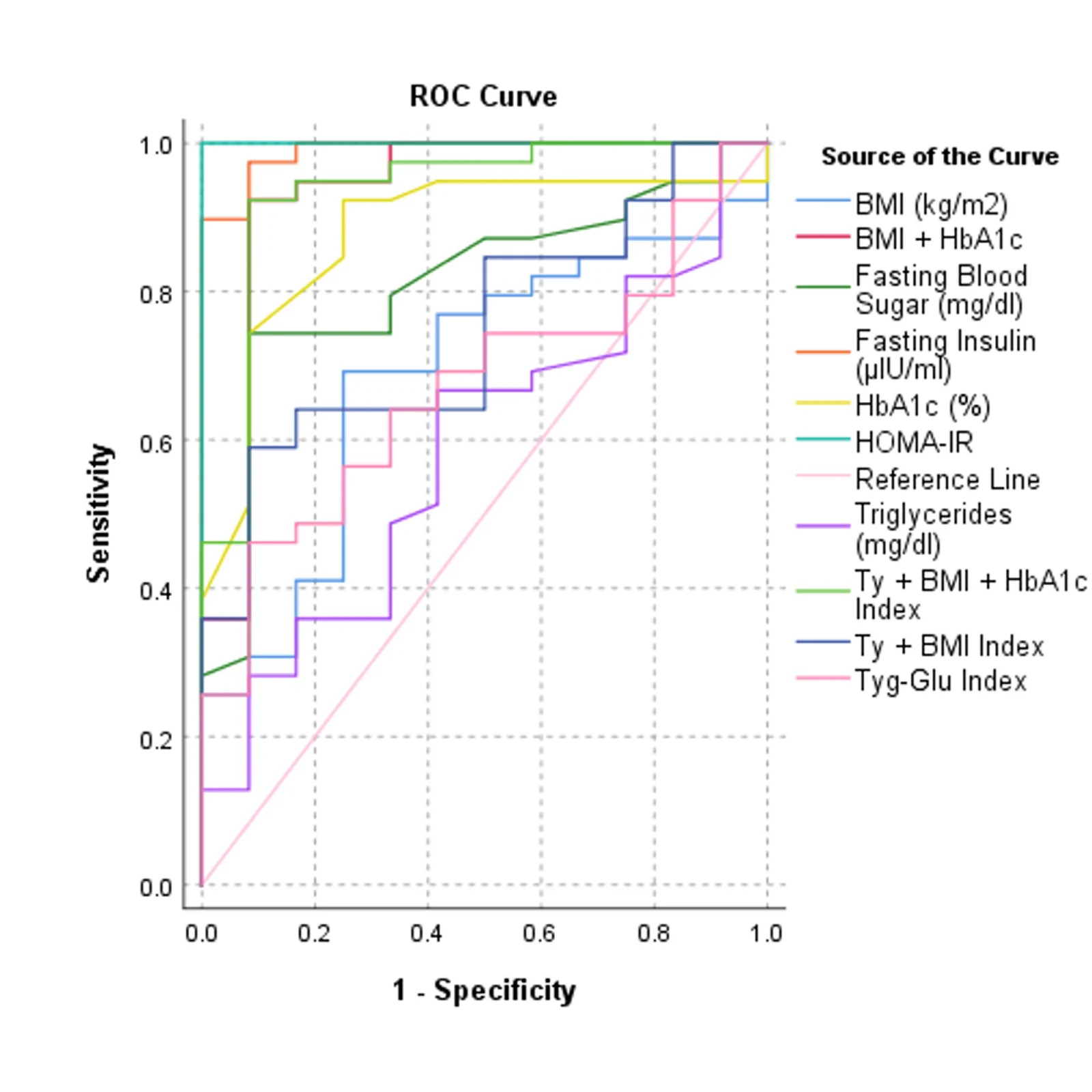
Diagnostic power of various parameters in the metabolic syndrome group. ROC: receiver operating characteristic; BMI: body mass index; HbA1c: glycated hemoglobin; HOMA-IR: Homeostasis Model Assessment of Insulin Resistance; TyG: triglyceride-glucose

ROC analyses demonstrated that insulin and HOMA-IR provided the strongest discrimination for insulin sensitivity, particularly in controls, pointing toward superior detection of primordial metabolic alterations. In MetS, both insulin-based measures and composite indices incorporating BMI and HbA1c showed robust discrimination, in line with the pathophysiology of metabolic dysfunction. Triglycerides showed consistently weak discriminatory performance across groups. These findings, illustrated in Figure 1 and Figure 2 and confirmed by AUC analysis, highlight the superiority of insulin-based and composite indices over isolated lipid markers, as shown in Table 7.

**Table 7:** Area under the curve analysis. AUC: area under the curve; BMI: body mass index; CI: confidence interval; HbA1c: glycated hemoglobin; HOMA-IR: Homeostatic Model Assessment for Insulin Resistance; TyG: triglyceride-glucose

Parameters	Overall		Controls		Cases	
	AUC (95% CI)	P-value	AUC (95% CI)	P-value	AUC (95% CI)	P-value
Fasting blood sugar (mg/dL)	0.699 (0.596–0.803)	<0.001	0.687 (0.523–0.85)	0.025	0.81 (0.679–0.941)	<0.001
Fasting insulin (µIU/mL)	0.972 (0.947–0.996)	<0.001	0.932 (0.865–0.998)	<0.001	0.989 (0.968–1.01)	<0.001
HOMA-IR	0.978 (0.957–0.999)	<0.001	0.941 (0.881–1.002)	<0.001	1 (1–1)	<0.001
Triglycerides (mg/dL)	0.572 (0.458–0.686)	0.215	0.635 (0.454–0.816)	0.143	0.585 (0.411–0.76)	0.338
BMI (kg/m²)	0.792 (0.704–0.88)	<0.001	0.663 (0.494–0.831)	0.058	0.688 (0.527–0.849)	0.022
HbA1c (%)	0.814 (0.726–0.902)	<0.001	0.536 (0.346–0.725)	0.712	0.878 (0.77–0.986)	<0.001
TyG index	0.798 (0.712–0.883)	<0.001	0.709 (0.544–0.874)	0.013	0.671 (0.516–0.826)	0.03
TyG + BMI index	0.829 (0.75–0.907)	<0.001	0.704 (0.544–0.865)	0.012	0.748 (0.606–0.89)	0.001
BMI + HbA1c	0.868 (0.794–0.941)	<0.001	0.651 (0.475–0.826)	0.092	0.932 (0.829–1.034)	<0.001
TyG + BMI + HbA1c index	0.874 (0.803–0.945)	<0.001	0.673 (0.501–0.844)	0.049	0.934 (0.844–1.023)	<0.001

The optimal cut-off study demonstrated that fasting insulin and HOMA-IR had the best sensitivity, specificity, and DORs across all groups, notably excelling in instances of MetS. Composite indices integrating BMI, HbA1c, and TyG components exhibited great specificity and robust DORs, affirming their use as dependable confirmatory diagnostic instruments. In contrast, triglycerides had low sensitivity and limited DOR values in all studies, suggesting inadequate standalone diagnostic efficacy. The data together indicate that insulin-based and composite metabolic indices provide enhanced diagnostic capability for classifying insulin sensitivity in comparison to isolated lipid measurements, as shown in Table 8.

**Table 8:** Analysis of diagnostic power. BMI: body mass index; DOR: diagnostic odds ratio; HbA1c: glycated hemoglobin; HOMA-IR: Homeostatic Model Assessment for Insulin Resistance; TyG: triglyceride-glucose

	Parameter	Cut-off	Sensitivity	Specificity	DOR
Overall	Insulin	>11.520	88.7	93.8	118
	HOMA-IR	>2.608	90.6	95.8	221
	Triglycerides	>184.500	47.2	77.1	3
	TyG index	>8.891	57.1	83.2	7
	BMI + HbA1c	>0.752	69.8	97.9	109
	TyG + BMI + HbA1c Index	>0.744	69.8	97.9	109
	TyG + BMI index	>0.391	79.2	75	11
Controls	Insulin	>7.790	100	80.6	114
	HOMA-IR	>1.837	100	83.3	136
	Triglycerides	>184.500	57.1	80.6	6
	TyG index	>8.554	50	65	2
	BMI + HbA1c	>0.384	35.7	94.4	9
	TyG + BMI + HbA1c Index	>0.338	50	86.1	6
	TyG + BMI index	>0.379	50	88.9	8
Metabolic syndrome	Insulin	>13.225	89.7	100	197
	HOMA-IR	>2.603	100	100	305
	Triglycerides	>161.600	66.7	58.3	3
	TyG index	>9.000	60	75	4
	BMI + HbA1c	>0.758	92.3	91.7	132
	TyG + BMI + HbA1c index	>0.744	92.3	91.7	132
	TyG + BMI index	>0.803	59	91.7	16

## Discussion

The study provides a thorough assessment of the diagnostic value of triglyceride-based indices and the HOMA-IR for detecting insulin resistance, as defined by QUICKI, in MetS. The study showed that insulin-based metrics, notably fasting insulin and HOMA-IR, consistently demonstrated greater efficacy in differentiating IR in both the general research population and the MetS subgroup. Furthermore, composite indices that include clinical and biochemical indicators, such as BMI and HbA1c, markedly improved diagnostic accuracy, particularly in those already exhibiting MetS. In contrast, isolated triglyceride levels and the TyG index had diminished discriminatory efficacy, especially when evaluated as independent indicators of IR in this pre-selected cohort. These findings emphasize the complex difficulties in precisely evaluating IR in metabolically impaired individuals and show the diverse diagnostic efficacy of several surrogate markers.

Our findings align with several studies from the Indian subcontinent and other LMICs, highlighting the significant incidence of MetS and the urgent need for adequate evaluation of IR [16]. Research from southern India revealed that HOMA-IR, the TyG index, and QUICKI exhibited good accuracy in identifying MetS, corroborating our observation of the strong efficacy of insulin-based metrics [16]. Likewise, another study in a South Asian cohort found that the HOMA-TG index had the largest AUC for diagnosing MetS, whereas QUICKI exhibited the lowest AUC in this setting, contrasting with our study’s reliance on QUICKI as a reference [23]. This gap indicates possible methodological differences or population-level traits that affect the comparative efficacy of different measures.

Nonetheless, several geographical studies provide divergent results for triglyceride-based indices. Our examination revealed little discriminating value for isolated triglycerides; nevertheless, a study from South India indicated that the TyG index surpassed HOMA-IR in forecasting T2DM [17,24]. These disparities may arise from population-specific variations in metabolic profiles, insulin dynamics, adiposity distribution, and the incidence of certain metabolic disorders. Research focusing on South Asians highlighted the difficulties arising from the absence of uniformity in insulin assays, which may restrict the clinical use of insulin-based surrogate indices [20]. Moreover, discrepancies in the incidence of isolated increased FPG compared to HbA1c in LMICs might confound diabetes diagnosis, affecting the interpretation of associated IR indicators [9]. The ethnic and regional variances highlight the need for region-specific validation of IR indicators to include unique genetic predispositions and lifestyle variables.

An incisive analysis of our results underscores the substantial diagnostic value of HbA1c, BMI, and composite indices, especially those that integrate these markers with triglycerides. HbA1c, indicative of prolonged glycemic exposure, accurately represents the enduring effects of IR on glucose management, making it a significant diagnostic element. BMI, as a measure of body fat, corresponds with the recognized association between surplus weight and IR. Triglycerides, integral to lipid metabolism, serve as physiologically relevant indicators due to the association of dyslipidemia with insulin resistance. Our findings indicate that composite indices, including the BMI + HbA1c and TyG + BMI + HbA1c Index, have been shown to be exceptional diagnostic indicators, exhibiting great specificity and strong DORs for QUICKI-defined IR in instances of MetS. The enhanced efficacy of these composite indices compared to individual indicators indicates a synergistic impact, whereby the amalgamation of many aspects of metabolic dysfunction, i.e., chronic glycemic exposure, obesity, and lipid metabolism, yields a more thorough and precise evaluation of IR. This discovery enhances existing knowledge by quantitatively confirming the supplementary diagnostic significance of composite measures, which have been theoretically acknowledged but inadequately substantiated for QUICKI-defined IR in MetS within an Indian population. The Single Point Insulin Sensitivity Estimator, which includes triglycerides, HDL, and BMI, has been suggested as a viable alternative to gold-standard tests in European populations; however, our study underscores the effectiveness of integrating anthropometric, glycemic, and lipid parameters for improved diagnostic accuracy in the local context [25].

The practical ramifications of these results are especially significant for resource-limited healthcare environments, notably in basic and secondary care in India. Conventional insulin-dependent metrics, such as HOMA-IR, often need specialized laboratory facilities, costly reagents for insulin tests, and meticulous pre-analytical procedures, which are sometimes inaccessible or prohibitively expensive in these contexts [25]. These constraints provide a substantial obstacle to the extensive screening and prompt detection of IR. In this context, composite indices that include easily measured factors such as HbA1c, BMI, and triglycerides provide a very practical and economical option. Triglyceride-based indices are emphasized as practical and dependable metrics confirmed against gold-standard methods, making them especially appropriate for resource-constrained nations [8,11]. These indicators use commonly accessible biochemical assays, reducing assay variability and logistical difficulties linked to more intricate evaluations. Affordable metabolic screening technologies correspond with current goals to expand diagnostic and risk assessment services in LMICs, enabling extensive screening campaigns without placing excessive financial strain on healthcare systems or individuals [21-23]. Our research highlights that although individual triglyceride levels may possess limited diagnostic value, their incorporation into meticulously designed composite indices with anthropometric and glycemic markers offers a practical and scalable method for screening IR and stratifying risk, promoting earlier intervention, and potentially alleviating the long-term impact of cardiometabolic diseases.

The merits of this research include its concentrated examination within a well-defined MetS cohort, enabling a precise evaluation of IR in a high-risk group. The stringent use of QUICKI as a recognized reference standard, based on fasting insulin and glucose levels, bolsters the credibility of our comparison research. Our results provide clinically interpretable insights into the efficacy of different IR indicators, providing practical advice for healthcare practitioners. Nevertheless, several restrictions need attention. The cross-sectional approach prevents the establishment of causation or the long-term predictive significance of these indicators for future cardiometabolic events. The external validity of our results may be affected by the particular demographic and clinical traits of our research cohort, thus restricting direct generalizability to other ethnic groups or larger populations. The dependence on surrogate indicators, while pragmatically essential, indicates that these metrics do not accurately represent the direct physiological processes of insulin action. Future research must focus on extensive prospective investigations among varied Indian and LMIC communities to corroborate these results and determine context-specific cut-off values for triglyceride-based and composite indicators. Incorporating these validated markers into comprehensive risk algorithms could markedly improve their predictive accuracy for clinical outcomes and guide the formulation of evidence-based policy guidelines for cost-effective screening and management of IR in resource-limited healthcare systems.

Strengths and limitations

The present study has several important strengths. It provides a comprehensive comparison of conventional insulin-based markers, triglyceride-based indices, and composite indices for the assessment of insulin resistance in an Indian population with MetS, using the QUICKI as the reference standard. Furthermore, the application of ROC curve analysis, multivariable logistic regression, and subgroup analyses enhanced the robustness of the diagnostic performance evaluation and strengthened the reliability of the study findings. However, certain limitations should be acknowledged. This was a single-center, cross-sectional study with a relatively modest sample size, which limits causal inference and may affect the generalizability of the findings to broader populations. Therefore, larger multicentric prospective studies are warranted to externally validate these findings before the proposed indices can be widely adopted in routine clinical practice.

## Conclusions

Our hypothesis stated that triglyceride-based indices would display diagnostic performance equivalent to HOMA-IR in diagnosing QUICKI-defined insulin resistance. However, our data reveal that standalone triglyceride-based indices often demonstrated worse discriminatory power relative to HOMA-IR. However, composite indices that include lipids, BMI, and HbA1c demonstrated strong diagnostic performance, making them a viable way to measure IR.

## References

[1] Ogbu ISI (2023). Criteria for the diagnosis of metabolic syndrome: a review. J Med Lab Diagn.

[2] Adeva-Andany MM, Martínez-Rodríguez J, González-Lucán M, Fernández-Fernández C, Castro-Quintela E (2019). Insulin resistance is a cardiovascular risk factor in humans. Diabetes Metab Syndr.

[3] Iglesies-Grau J, Garcia-Alvarez A, Oliva B (2023). Early insulin resistance in normoglycemic low-risk individuals is associated with subclinical atherosclerosis. Cardiovasc Diabetol.

[4] Zhang A, Huang L, Tang M (2024). Non-linear associations of HOMA2-IR with all-cause mortality in general populations: insights from NHANES 1999-2006. BMC Public Health.

[5] Sasaki N, Ueno Y, Higashi Y (2024). Indicators of insulin resistance in clinical practice. Hypertens Res.

[6] Antoniolli LP, Nedel BL, Pazinato TC, de Andrade Mesquita L, Gerchman F (2018). Accuracy of insulin resistance indices for metabolic syndrome: a cross-sectional study in adults. Diabetol Metab Syndr.

[7] Alkhaldy AA, Rizq NK, Del Jaylan SA, Alkendi EA, Alghamdi WM, Alfaraidi SM (2019). Dietary intake and physical activity in relation to insulin resistance in young overweight Saudi females: an exploratory pilot study. Prev Nutr Food Sci.

[8] Tahapary DL, Pratisthita LB, Fitri NA (2022). Challenges in the diagnosis of insulin resistance: focusing on the role of HOMA-IR and tryglyceride/glucose index. Diabetes Metab Syndr.

[9] Khan SH, Sobia F, Niazi NK, Manzoor SM, Fazal N, Ahmad F (2018). Metabolic clustering of risk factors: evaluation of Triglyceride-glucose index (TyG index) for evaluation of insulin resistance. Diabetol Metab Syndr.

[10] Kurniawan LB (2024). Triglyceride-glucose index as a biomarker of insulin resistance, diabetes mellitus, metabolic syndrome, and cardiovascular disease: a review. EJIFCC.

[11] Gounden V, Devaraj S, Jialal I (2024). The role of the triglyceride-glucose index as a biomarker of cardio-metabolic syndromes. Lipids Health Dis.

[12] Avagimyan A, Pogosova N, Fogacci F (2025). Triglyceride-glucose index (TyG) as a novel biomarker in the era of cardiometabolic medicine. Int J Cardiol.

[13] Park HM, Lee HS, Lee YJ, Lee JH (2021). The triglyceride-glucose index is a more powerful surrogate marker for predicting the prevalence and incidence of type 2 diabetes mellitus than the homeostatic model assessment of insulin resistance. Diabetes Res Clin Pract.

[14] Gao W, Deng Z, Gong Z, Jiang Z, Ma L (2025). AI-driven prediction of insulin resistance in non-diabetic populations using minimal invasive tests: comparing models and criteria. Diabetol Metab Syndr.

[15] Prashant A, Nataraj SM, Swetha N (2024). Unveiling the significance of surrogate markers of insulin resistance in metabolic health assessment. Indian J Med Biochem.

[16] Endukuru CK, Gaur GS, Yerrabelli D, Sahoo J, Vairappan B (2020). Cut-off values and clinical utility of surrogate markers for insulin resistance and beta-cell function to identify metabolic syndrome and its components among Southern Indian adults. J Obes Metab Syndr.

[17] Jog KS, Eagappan S, Santharam RK, Subbiah S (2023). Comparison of novel biomarkers of insulin resistance with Homeostasis Model Assessment of Insulin Resistance, its correlation to metabolic syndrome in South Indian population and proposition of population specific cutoffs for these indices. Cureus.

[18] Hu L, Zhang Q, Bai Y, Hu G, Li J (2022). Triglyceride-glucose index correlate with telomere length in healthy adults from the National Health and Nutrition Examination Survey. Front Endocrinol (Lausanne).

[19] Wan Y, Zhang Z, Ling Y (2023). Association of triglyceride-glucose index with cardiovascular disease among a general population: a prospective cohort study. Diabetol Metab Syndr.

[20] Fosam A, Bansal R, Ramanathan A (2023). Lipoprotein insulin resistance index: a simple, accurate method for assessing insulin resistance in South Asians. J Endocr Soc.

[21] Sathish T, Shaw JE, Tapp RJ, Wolfe R, Thankappan KR, Balachandran S, Oldenburg B (2019). Targeted screening for prediabetes and undiagnosed diabetes in a community setting in India. Diabetes Metab Syndr.

[22] Sivaprasad S, Raman R, Rajalakshmi R (2020). Protocol on a multicentre statistical and economic modelling study of risk-based stratified and personalised screening for diabetes and its complications in India (SMART India). BMJ Open.

[23] Mirr M, Skrypnik D, Bogdański P, Owecki M (2021). Newly proposed insulin resistance indexes called TyG-NC and TyG-NHtR show efficacy in diagnosing the metabolic syndrome. J Endocrinol Invest.

[24] Bazyar H, Zare Javid A, Masoudi MR (2024). Assessing the predictive value of insulin resistance indices for metabolic syndrome risk in type 2 diabetes mellitus patients. Sci Rep.

[25] Dudi P, Goyal B, Saxena V (2019). Single point insulin sensitivity estimator as an index for insulin sensitivity for metabolic syndrome: a study in North Indian population. J Lab Physicians.

